# Re-evaluation of the methodology for estimating the US specialty physician workforce

**DOI:** 10.1093/haschl/qxae033

**Published:** 2024-03-19

**Authors:** W Stephen Black-Schaffer, David J Gross, Zakia Nouri, Aidan DeLisle, Michael Dill, Jason Y Park, James M Crawford, Michael B Cohen, Rebecca L Johnson, Donald S Karcher, Thomas M Wheeler, Stanley J Robboy

**Affiliations:** Department of Pathology, Massachusetts General Hospital, and Harvard Medical School, Boston, MA 02114, United States; Policy Roundtable, College of American Pathologists, Washington, DC 20001, United States; Workforce Studies, Association of American Medical Colleges, Washington, DC 20001, United States; Policy Roundtable, College of American Pathologists, Washington, DC 20001, United States; Workforce Studies, Association of American Medical Colleges, Washington, DC 20001, United States; Department of Pathology, University of Texas Southwestern Medical Center, Dallas, TX 75390, United States; Department of Pathology and Laboratory Medicine, Northwell Health, New Hyde Park, NY 11040, United States; Department of Pathology, Wake Forest University, Winston-Salem, NC 27109, United States; American Board of Pathology (retired), Tampa, FL 33609, United States; Department of Pathology, The George Washington University School of Medicine and Health Sciences, Washington, DC 20052, United States; Department of Pathology and Immunology, Baylor College of Medicine, Houston, TX 77030, United States; Duke Pathology, Duke University School of Medicine, Durham, NC 27710, United States

**Keywords:** physician workforce, physician supply, pathology, AMA Physician Professional Data, subspecialization

## Abstract

Increasing pursuit of subspecialized training has quietly revolutionized physician training, but the potential impact on physician workforce estimates has not previously been recognized. The Physicians Specialty Data Reports of the Association of American Medical Colleges, derived from specialty designations in the American Medical Association (AMA) Physician Professional Data (PPD), are the reference source for US physician workforce estimates; by 2020, the report for pathologists was an undercount of 39% when compared with the PPD. Most of the difference was due to the omission of pathology subspecialty designations. The rest resulted from reliance on only the first of the AMA PPD's 2 specialty data fields. Placement of specialty designation in these 2 fields is sensitive to sequence of training and is thus affected by multiple or intercalated (between years of residency training) fellowships. Both these phenomena have become progressively more common and are not unique to pathology. Our findings demonstrate the need to update definitions and methodology underlying estimates of the US physician workforce for pathology and suggest a like need in other specialties affected by similar trends.

## Introduction

Accurate physician specialty supply estimates are crucial for determining current and future workforce needs. The Association of American Medical Colleges' (AAMC’s) Physician Specialty Data Report (Data Report)^1^ is a key source of standardized, consistently published estimates of physician supply in the United States, both overall and by specialty. The AAMC's Data Report has been cited in congressional reports and testimony.^2-5^ Data on practicing physicians featured in this Data Report are largely derived from the American Medical Association (AMA) Physician Professional Data (PPD; formerly known as the AMA Masterfile). The PPD began in 1906 as a membership and mailing directory and evolved into a database of 1.4 million US physicians, residents, and medical students.^6^ The AAMC's methodology is described elsewhere.^1^

In a recent pathology workforce study,^7^ a comparison of the 2018 Data Report^8^ and the 2019 AMA PPD provided by the AMA to the College of American Pathologists showed pathologist numbers of 12 839 and 21 292, respectively.^7^ The source of this discrepancy lay in the PPD's operationalized definition of pathologists, which counted physicians as pathologists only when 1 of 4 specialty designations appeared in the AMA's practice specialty field as the primary specialty,^9^ whereas the PPD tracks 15 general and subspecialty pathology designations across both primary and secondary specialty data fields. The combination of the AAMC's narrow specialty definition and the changing character of graduate medical education in pathology (specifically the increasing prevalence of subspecialty training) underlies this discrepancy in the counts of US pathologists.^7^

The AAMC partnered with the College of American Pathologists (CAP) to explore and address this discrepancy, by updating the operationalized definition applied by the AAMC in identifying which physician designations in the AMA PPD should be counted as pathologists, and accounting for these designations using both the primary and secondary AMA PPD specialty data fields.^9^ While there are other sources for estimating pathology supply, such as claims data or state licensure data, each has shortcomings that are more problematic than those associated with the PPD. Most of the correction in overall pathologist count was due to the comprehensive recognition of pathology-specific specialty designations, with the residue of the correction due to consideration of both specialty designation fields. Features of pathology training that may influence placement of specialty and subspecialty designations between these 2 specialty data fields are multiple fellowships and fellowships intercalated between years of residency training. Through this “case study” of pathology, insights gleaned from these improved methods of estimation may have broader applicability to the assessment of other physician specialties for the purposes of supply estimation.

This paper focuses solely on how to obtain more accurate estimates of the supply of a physician specialty, focusing on the example of pathology. While we recognize the importance of developing a strong methodology for estimating demand for specialty services,^10^ we contend that any such analysis requires a reliable and agreed-upon methodology for understanding what physicians are in that specialty. Further, this paper does not purport to address scope-of-practice issues among different subspecialties of pathology or relative supply between subspecialty areas, as such issues are beyond the scope of our paper.

## Data and Methods

This study used AMA physician data spanning 2004–2020, including age, sex, and specialty fields (AMA tracks 2 specialty fields, designated as “primary” and “secondary”) and linked to the AAMC's race/ethnicity data. (AAMC/AMA PPD data collection for a given year closes on December 31 and is subsequently reported at the beginning of the next year; as a result, data contained in an annual report are lagged 1 year. So, for example, the 2005 report, and associated variable label in the PPD, contains the 2004 data.) Two aspects of this primary data were considered in this study.

First was identification of those specialty designations that indicate the physician is a pathologist. The legacy AAMC method for counting pathologists from the AMA PPD has been confined to only 4 specific pathology designations—Anatomic Pathology (ATP), Clinical Pathology (CLP), Chemical Pathology (PCH), and Pathology—with this last designation signifying Anatomic/Clinical Pathology (PTH). A panel of pathologists serving on the CAP Policy Roundtable's Workforce and Graduate Medical Education Workgroup reviewed all specialty designations appearing in either of the 2 AMA specialty fields between 2004 and 2020 in which there was a code for at least 1 of the 15 pathology designations recognized by the American Board of Pathology (ABPath) or the Accreditation Council for Graduate Medical Education (ACGME) as a type of certification or accredited training in a pathology specialty or subspecialty. These 15 codes correspond to 3 residency training combinations (Anatomic Pathology [ATP], Clinical Pathology [CLP], Anatomic/Clinical Pathology [PTH]) and 12 pathology subspecialty fellowships (Blood Banking [BBK]/Transfusion Medicine, Chemical Pathology [PCH], Clinical Informatics [Pathology] [CIP]; Cytopathology [PCP]; Dermatopathology [DMP]; Forensic Pathology [FOP]; Hematology Pathology [HMP]; Medical Microbiology [MM]; Molecular Genetic Pathology [MGP]; Neuropathology [NP]; Pediatric Pathology [PP]; and Selective Pathology [SP]).

The second aspect of these primary data that we evaluated was their derivation from the 2 specialty data fields in the AMA PPD. The legacy AAMC method for counting physicians from the AMA PPD had been to consider only the primary specialty data field. This approach implicitly assumes that the first AMA data field corresponds to the physician's initial specialty training, and hence presumptively to residency or “primary” training in the colloquial sense. The second data field is likewise assumed to correspond to subsequent training, presumptively in a subspecialty of the physician's area of residency training. (It should be noted that physicians are able to manually update the primary and secondary specialty fields in the AMA database; however, we have no information on how many physicians take advantage of this opportunity.) Our comprehensive review of all of the actual combinations of primary and secondary listed specialty designations, however, showed no discernible pattern of specialty and subspecialty listings that might support these assumptions about the AMA data fields. In Table 1, the left half shows examples of actual first (“primary”) and second (“secondary”) specialty data field combinations that are found in the AMA PPD. To minimize confusion with common parlance, in which primary might be taken to mean residency training and secondary to mean subsequent or fellowship-based subspecialty training, these “primary” and “secondary” fields will henceforth be referred to as the first and second specialty data fields, respectively, without further connotation.

**Table 1.** Examples of specialty data fields from AMA Physician Professional Data.

Examples of specialty data fields from AMA masterfile			Three new options for counting pathologists using specialty data		
Group	Specialty field 1 (primary)	Specialty field 2 (secondary)			
**A**	Other Specialty	Clinical Pathology	Narrowest definition = Group A	Groups A and B	Broadest definition = groups A, B, and C
	Pathology	Clinical Pathology			
	Anatomic Pathology	Cytopathology			
	Pathology	Dermatopathology			
	Pathology	Unspecified			
	Clinical Pathology	Other Specialty			
	Cytopathology	Pathology			
	Neuropathology	Clinical Pathology			
	Forensic Pathology	Dermatopathology			
	Dermatopathology	Unspecified			
	Pediatric Pathology	Other Specialty			
**B**	Pathology	Internal Medicine			
	Pathology	Oncology			
	Clinical Pathology	Infectious Diseases			
	Dermatopathology	Dermatology			
	Pathology	Obstetrics/Gynecology			
**C**	Dermatology	Pathology			
	Dermatology	Dermatopathology			
	Internal Medicine	Anatomic Pathology			
	Neurology	Neuropathology			
	Obstetrics/Gynecology	Pathology			

Abbreviation: AMA, American Medical Association.

Utilizing these comprehensive 15 pathology designations, consideration of their listing in the 2 data fields provided 3 ways to count physicians who might be considered to be pathologists, as follows:

- Group A includes individuals with any of the 15 pathology designations in either or both specialty data fields, but excludes anyone with a specific non-pathology designation in either data field. This definition and methodology yield the most specific possible pathology combinations using these 15 pathology designations. In that it excludes anyone with an indication of specific non-pathology training, this methodology potentially excludes some physicians with specific non-pathology training who may nevertheless actually practice pathology.
- Group B differs from group A in that, like the legacy AAMC method, it considers only the first (“primary”) data field. This corresponds to changing only the listed specialty definitions from the previous methodology. It will therefore include individuals with specific non-pathology designations in the second field.
- Complementing the 2 previous groups to comprise the broadest possible count of pathologists, group C is those physicians who have a pathology designation in the second data field and a specific non-pathology designation in the first data field. The combination of groups A, B, and C thus constitutes the broadest, although least specific, possible count of pathologists: while it is most likely to capture anyone in the dataset who might practice pathology, it also is most likely to include physicians whose underwent pathology training but whose actual practice is in a non-pathology specialty.

Combinations of these 3 groups thus provide a perspective on the full range of possible methods, from the narrowest and most specific to the broadest but least specific methodology for counting pathologists using these 15 pathology specialty designations. For the purposes of this report, we tested 3 options for counting a “pathologist” (shown in the right-hand side of Table 1).

- The first option changes the criteria for which specialty designations should be counted for a pathologist, expanding AAMC's legacy 4 designations above to include physicians with any 1 of the 15 pathology designations, and makes the additional methodological change of including physicians whose records show 1 of these 15 pathology designations in the first and/or second data fields, while excluding physicians with a specific non-pathology designation in either field. This is group A in Table 1.
- The second option changes only the criteria for which specialty designations should be counted for a pathologist, expanding AAMC's legacy 4 designations above to include physicians with any 1 of the 15 pathology designations in the first specialty data field. Referring to Table 1, this second option represents physicians in groups A and B. (Note that it does not include the small number of physicians in group A with no specific designation in the first data field and a pathology designation in the second data field.)
- The third option, presented for comparison purposes, includes physicians whose records show 1 of the 15 pathology designations in the first and/or second data fields, but does not exclude those who also have a non-pathology specialty designation.

Statistics and analyses were conducted using SAS Enterprise 8.3 (SAS Institute Inc., Cary, NC, USA).

## Results

### Pathologist supply

**Figure 1.**
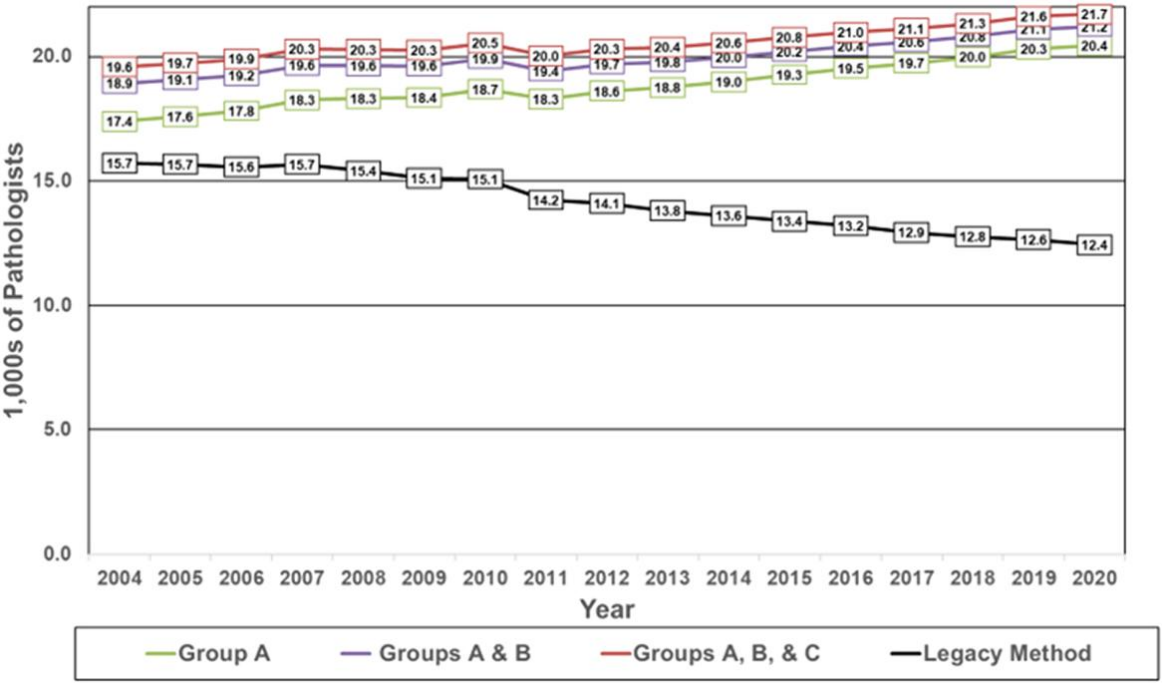
Comparison of estimates of pathologist supply in the United States using the legacy method (AAMC reports) and groups A, B, and C: 2004–2020. Abbreviation: AAMC, Association of American Medical Colleges.

We calculated alternative estimates for pathologist supply from 2004—when AAMC records were first digitally available—to 2020. These estimates reflect the 3 potential definitions, described in Table 1, for which physicians in the AMA PPD should be counted as pathologists. Figure 1 shows that, for every year, the alternative definitions for US pathologist workforce resulted in higher numbers of pathologists than those appearing in the AAMC Data Reports. In 2004, the AAMC legacy method used for counting the pathologist workforce, which included only physicians with designation of Anatomic Pathology, Clinical Pathology, Anatomic Pathology/Clinical Pathology (PTH), or Chemical Pathology (PCH) listed in the first specialty data field, reported the number of pathologists as 15 700. This AAMC approach has reported an incremental yearly decrease in pathologists nearly every year since, with reported pathologist supply falling to 12 400 in 2020 (black line in Figure 1).

The purple line in Figure 1 (groups A and B) isolates the impact of only changing the criteria for which specialty designations should be counted as a pathologist (ie, counting all 15 pathology specialty designations) but not changing the methodology of looking only at the first specialty data field. Including physicians who appear in groups A and B, the pathologist count in 2004 was 18 900, progressively rising to 21 200 in 2020, thus differing from counts in the AAMC Data Reports by 17% in 2004, rising to 42% by 2020 (purple line in Figure 1), corresponding to the increasing prevalence of subspecialty training over that time.

However, as previously noted, our review of all the actual combinations of first and second listed specialties in the AMA PPD shows no basis for limiting the count to the first specialty data field. The green line in Figure 1 shows the impact of (1) assessing both data fields for the 15 pathology designations and (2) excluding those physicians with a specific non-pathology designation in 1 of the fields (ie, those who might reasonably be thought to be practicing in a different specialty). Referring to Table 1, this corresponds to group A. Using this approach, the estimated number of pathologists in 2004 was 17 400 (1500 fewer pathologists than under the purple line in Figure 1), progressively rising in 20 400 in 2020 (800 fewer pathologist than under the purple line in Figure 1), differing from counts in the corresponding AAMC Data Report by 10% in 2004, rising to 39% in 2020 (green line in Figure 1).

We contend that using only group A provides the least ambiguous approach for counting the number of pathologists, as it includes all 15 pathology specialty designations while excluding physicians who may be practicing in (and therefore might plausibly be counted in) a non-pathology specialty; and, in fact, the number of physicians who appear only in group B or group C is small and decreasing, and does not substantially change our findings. As shown by the purple line in Figure 1, in the most recent year for which we have data, including all 15 pathology designations accounted for an additional 8800 pathologists compared with the legacy AAMC method. By contrast, including these same 15 specialty designations but also considering both specialty data fields excludes a net number of 800 physicians for a count of 8000 over the AAMC estimate. So, to avoid possible ambiguity of physician assignment if this method of considering both data field designations were to be generalized to other physician specialties, only group A data are considered in the analyses in the remainder of this report.

### Growth in subspecialty training

**Figure 2.**
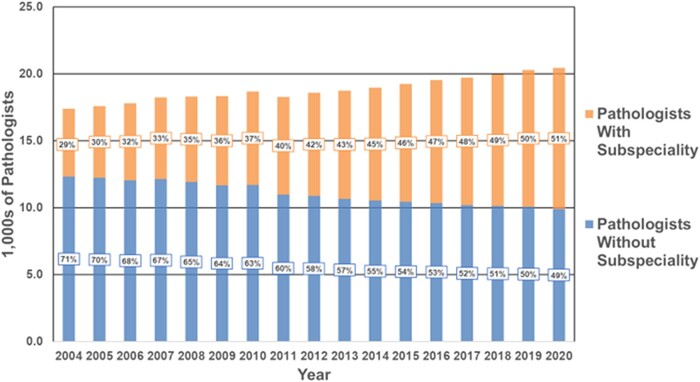
Trends in pathology supply, pathologists without subspecialty training vs pathologists with subspecialty training, 2004-2020. “Legacy Method” refers to the method used to date to count pathologists, as reported in AAMC Data Report (ie, counting only pathologists for whom anatomic pathology, clinical pathology, anatomic/clinical pathology, or chemical pathology appears in the first data field). Abbreviation: AAMC, Association of American Medical Colleges.

Using group A data, Figure 2 shows the increasing percentage of pathologists in the AMA PPD who have trained in a pathology subspecialty, rising from 29% in 2004 to 51% in 2020.

### Pathologist age

**Figure 3.**
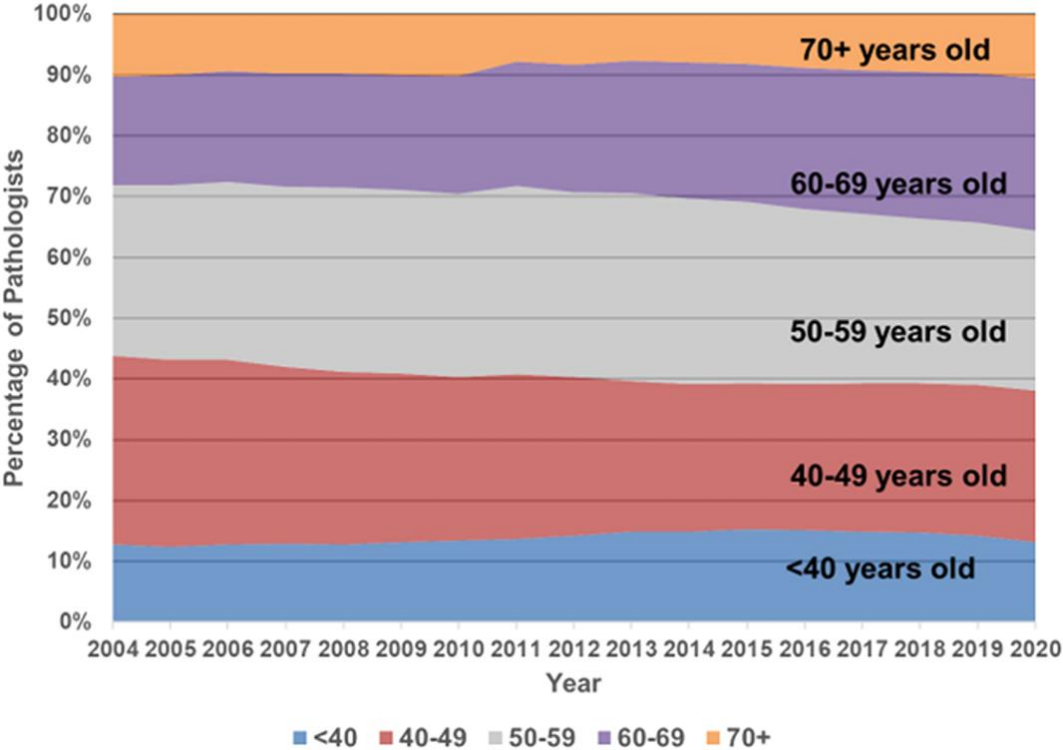
Distribution of pathologists by age cohorts, 2004–2020.

Grouping the pathologist workforce into 5 age cohorts (<40 years, 40–49 years, 50–59 years, 60–69 years, ≥70 years) (Figure 3) disclosed that the total numbers in the 3 younger groups have remained roughly constant. As members of the 50–59 cohort (grey band in Figure 3) age into the 60–69 cohort (purple band), they are being replaced by smaller numbers of the 40–49 cohort (red band) aging into the 50–59 cohort, resulting in a substantial and growing disproportion of pathologists in the oldest two decades of practice.

**Figure 4.**
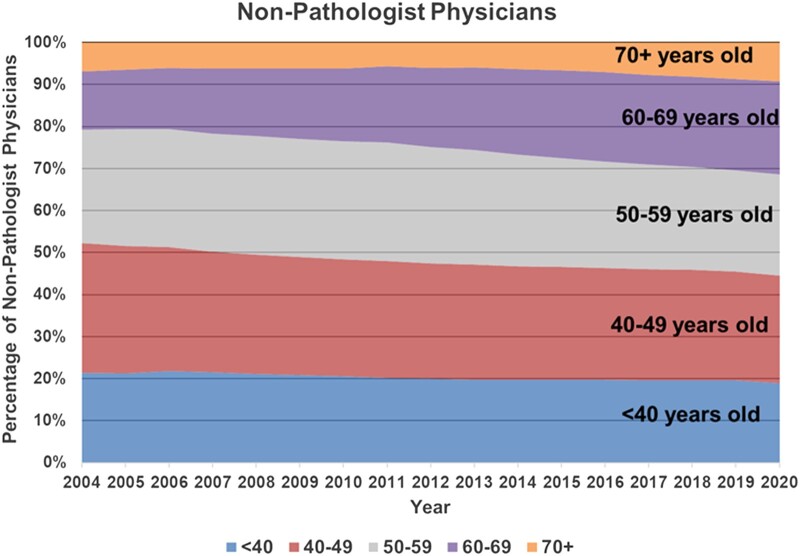
Distribution of non-pathologist physicians by age cohorts, 2004–2020.

This proportionate decrease in young and middle-aged (<60 years) pathologists shows there is an insufficient number of these pathologists to replace the older pathologists as they age out of practice. Specifically, the percentage of pathologists in the age 60–69 cohort (Figure 3, purple band) increased substantially, from 17.9% in 2004 to 25.0% in 2020. Meanwhile, the percentage of pathologists under age 40 (Figure 3, blue band) increased as well, but only slightly, from 12.7% in 2004 to 13.2% of active US pathologists in 2020. Furthermore, pathologists aged 50–69 years represented over one-half of active US pathologists in 2020 (51.3%; gray and purple bands in Figure 3). As the cohorts in the gray and purple bands age over the coming years, many will leave pathology practice due to retirement, mortality, or other reasons (commonly, shifting to non-practicing roles). As these older cohorts of pathologists leave practice (demonstrated by the consistently narrower size of the orange band of pathologists over age 70), there will be fewer pathologists in the ≤40- and 40–49-year cohorts to replace them. This may also be an issue among non-pathologist physicians, but the problem is proportionately greater among pathologists. Specifically, while the share of non-pathologist physicians aged 60–69 years increased substantially from 2004 (13.9%) to 2020 (22.1%), and in 2020 is slightly higher than the cohort of non-pathologist physicians under age 40 (18.9%, down from 21.3% in 2004), the age-60–69-year and under-age-40-year groups of non-pathologist physicians each represent roughly one-fifth of practicing non-pathologist physicians. (See Figure 4 for the distribution of non-pathologist physicians by age cohorts from 2004 through 2020.) This rough equivalence (each about 1/5) among non-pathologist physician of the 60–69 and under-40 age groups^11^ contrasts sharply with the demographics of pathologists, where the 60–69 age group (at 25.0%) is almost double the size of the under age 40 group (at 13.2%).

### Composition of pathologist supply by sex

Given that more than one-third of today's US physicians are women, and women currently comprise nearly half of all resident-trainees the United States,^10,12^ we sought to discover what our revised pathologist demographics tells us about the gender composition of pathology. Here, too, the revised method for counting pathologists proposed in this paper reveals a distinctly different picture, showing that an increasing percentage of recent entrants to the pathology workforce are women. Under the legacy method, the number of women pathologists appeared roughly constant (4687 in 2004 and 4811 in 2020). However, our revised count shows that the number of woman pathologists has risen substantially, from 5532 in 2004 to 8864 in 2020. In contrast, the number of pathologists who are men has remained essentially constant (11 853 in 2004 and 11 558 in 2020).

### Racial and ethnic composition of pathologist supply

Finally, we sought to better understand the changing racial and ethnic makeup of the pathologist workforce over time. The demographic shifts in the pathologist workforce largely mirror those of the non-pathologist physician workforce, although there are a few notable points of divergence, as follows:

- The percentage of pathologists who identify as Black or African American in 2004 was approximately half that of non-pathologist physicians: 1.8% of pathologists vs 3.5% of non-pathologist physicians. In 2020, the percentage of pathologists who identify as Black or African American remained just over half that of non-pathologist physicians, with each at a somewhat higher level: 2.9% of pathologists self-identified as Black or African American vs 5.1% of non-pathologist physicians.
- The percentage of pathologists who identify as Hispanic increased from 3.6% in 2004 to 5.9% in 2020. Non-pathologist physicians saw the same 2.3% increase in Hispanic representation that pathologists did, rising from 3.8% in 2004 to 6.1% in 2020.
- The percentage of pathologists who identify as Asian grew substantially for all physicians, but it has been—and continues to be in the 2020 data—somewhat higher for pathologist than for non-pathologist physicians. The share of pathologists who identified as Asian increased from 9.1% in 2004 to 19.5% in 2020, while the corresponding shares for non-pathologist physicians were 8.2% in 2004 and 17.9% in 2020.
- Physicians who identify as White continue to make up similar majorities of both the pathologist and non-pathologist physician workforce. Among pathologists, 50.5% identified as White in 2004 and 58.6% did so in 2020. Comparatively, 50.3% non-pathologist physicians identified as White in 2004 and 56.4% did so in 2020.

The largest single shift between 2004 and 2020 has been in the “Unknown” category: the percentage of physicians who have declined to provide identifying information or for whom racial and ethnic data were not collected has fallen from 33.8% to 10.4% for pathologists and from 33.3% to 12.1% of non-pathologist's physicians. While this would appear to represent a significant improvement in the collection rate of potentially meaningful ethnicity and racial information for all physicians over time, it also confounds our ability to draw conclusions about physician demographic changes over time, as it is unclear to what extent the preceding apparent increase in the percentage of non-White physicians is because physicians have increasingly chosen to identify themselves as such, and to what extent it reflects actual changes in the physician workforce.

## Discussion

The AAMC's specialty-specific physician workforce estimates serve the workforce research community, physician specialty societies, and policymakers seeking detailed data on how many and what types of physicians are in practice, and thus how many more may be needed to meet the nation's medical care needs. Accordingly, how any given specialty is defined, specifically who will be counted and who will not, can affect policy decisions in multiple ways. Maintaining up-to-date definitions is critical to accurately informing public policy decisions about the physician workforce, as well as to medical student career choices and training program resource allocations. With over 200 extant physician specialties, the AAMC relies on the physician specialty and subspecialty societies to work with them to maintain current definitions.

Our findings confirm that the current definition and methodology underlying publicly available estimates of the US pathologist workforce needs to be updated to avoid confounding due to the increasing prevalence of subspeciality training in the pathologist workforce. In 2004 (the earliest year for which AMA PPD were available for analysis in this study), the legacy method resulted in a count 10% lower than the revised method, with this disparity growing to 39% in 2020 (using our least ambiguous approach for counting pathologists). A smaller but important correction to the revised count is attributable to the methodological change which considers both physician specialty data fields in the AMA PPD rather than just the first data field.

Furthermore, these data confirm a long-expected demographic trend in pathology associated with the approximately 25% decrease in the number of pathology residency training positions in the United States since the early 1990s.^13,14^ Despite the overall non-pathologist less-than-40-year physician cohort being somewhat smaller than the other age groups—suggesting a potential future physician shortage in at least some other specialties—this less-than-40-year physician shortfall is greater in pathology. Even including pathologists up through age 49, pathology has a lower proportion of physicians in the younger cohorts than other physicians (38.1% of pathologists in 2020 compared to 44.5% of non-pathologist physicians in the same year). Correspondingly, pathology is “top heavy” in older physicians, contributing to a pathologist employment market that is already showing signs of supply shortage.^10^

A consequence of an improved method for estimating pathologist supply is a more accurate reporting of trends in the specialty's demographics, particularly since younger pathologists are more likely to be subspecialty trained. Indeed, well over 95% of today's trainees are subspecialty trained or intend to have subspecialty training, and a majority of them are women.^12^

Thus, in this paper, we propose an updated definition of pathologists and a revised methodology for counting them, which, between them, provides a more accurate estimate of the current pathology workforce. Specifically, we propose that all physicians for whom a pathology specialty or subspecialty designation is listed in the AMA PPD be counted, while excluding those who also list a specific non-pathology designation as being of ambiguous specialty. We also contend that the enumerations of how many physicians are in a particular specialty should examine both the first and second data field in the AMA PPD (rather than solely the first data field). Our approach was to include physicians who had a pathology-related designation in either data field, so long as neither field listed another specific physician specialty designation. Finally, for the purposes of trending, we recommend that this methodology be applied to the AMA PPD for pathologist workforce estimates dating back to 2004. This will help ensure that attempts to understand and make policy based on the evolving demographics of the specialty are well founded.

While this report relates specifically to pathology, we believe it has implications for other medical specialties. The AMA PPD lists 28 specialties/major groupings with 265 subspecialties.^9^ More than 80% of surgical residents plan to subspecialize,^15^ which may lead to a paradigm shift in the practice of surgical medicine.^16^ According to data from the National Residency Matching Program (NRMP), 32% of all current first-year postgraduate trainees enter subspecialty fellowship positions.^17,18^ For pediatrics, it is 51%; for internal medicine, 66%; and it is 61% for surgery. While these NRMP numbers may not be reflected in all subspecialties, the trend toward subspecialty training among graduating physicians is clear. The NRMP provides an early indicator of workforce trends as these data are public even before physicians enter their graduate training.^19^ If, as in pathology, some subspecialty designations are not being captured by current definitions, then the workforce estimates in those specialties may also be misstated. At the very least, specialty-specific estimates of the physician workforce need to ensure that they account for growing numbers of physicians with training in 1 or more subspecialty areas.

Of consequent concern is the potential for specialty-related policy decisions affecting the physician workforce to be mistargeted.^20^ This could include funding for graduate medical education training slots and redirecting of health care resources based on perceived physician shortages in a given specialty. Inaccurate estimates suggesting an incipient dearth or glut of physicians in any specialty may profoundly influence decision making by undergraduate physicians-in-training regarding selection of their categorical specialty, as these raise concerns, respectively, about the attractiveness of practice or the availability of positions in that specialty.

A potential limitation of this work revolves around the source of data used to populate the AMA PPD.^21^ While much is automatically populated by data collection from training programs to support FREIDA, the AMA Residency & Fellowship Database, physicians also can freely edit their own listings.^22^ Separately, auto-population may be affected both by multiple and by intercalated fellowships (fellowships taken between years of residency training). Both these phenomena are common in pathology, possibly contributing to the substantial variability in whether categorical residency training appears in the first specialty data field of the AMA PPD, or in some cases not at all. Although both these phenomena are common in pathology, neither is unique to pathology.

Additionally, while accompanying data for age and sex are well populated, the data available for this study lacked completeness for race and ethnicity. The data are confounded by a large—albeit decreasing—number of observations for which race/ethnicity data are unknown. This study thus cannot draw definitive conclusions about trends in race and ethnicity for pathology as a specialty.

This study represents a collaboration between the AAMC and the CAP. This collaboration has been essential, and our findings have prompted a re-evaluation of the operational definition for pathology as well as the general methodology for future AAMC workforce reporting. Other medical specialty organizations may want to consider the need for assessment of their physician workforce estimates, and engage with the AAMC to examine available data. There are potentially profound implications for communication with physicians-in-training at the undergraduate and graduate levels, resourcing of training programs at the graduate level, and deployment of physicians across the United States. Having accurate estimates of the physician workforce will also be important for communicating with the public as regards the US health care system's ability to meet society's ongoing needs.

## Disclaimer

While Dr. Gross and Mr. DeLisle are employed by the College of American Pathologists (CAP), the opinions expressed here do not necessarily reflect the views of the CAP.

## Supplementary Material

qxae033_Supplementary_Data
